# 25-Hydroxy Vitamin D Deficiency Is Associated With Cardiovascular Sequential Organ Failure Assessment and Pediatric Risk of Mortality III Scores in Critically Ill Children

**DOI:** 10.3389/fped.2020.00066

**Published:** 2020-02-28

**Authors:** Hongxing Dang, Jing Li, Chengjun Liu, Feng Xu

**Affiliations:** ^1^Department of PICU, Ministry of Education Key Laboratory of Child Development and Disorders, Children's Hospital of Chongqing Medical University, Chongqing, China; ^2^National Clinical Research Center for Child Health and Disorders, China International Science and Technology Cooperation Base of Child Development and Critical Disorders, Chongqing, China; ^3^Chongqing Key Laboratory of Child Health and Nutrition, Chongqing, China

**Keywords:** 25-hydroxyvitamin D, severity of illness index, critical illness, intensive care, children

## Abstract

**Aim:** Investigate 25-hydroxy vitamin D (25(OH)D) levels and the correlation with cardiovascular sequential organ failure assessment (CV-SOFA) and pediatric risk of mortality III (PRISM-III) scores in critically ill children.

**Methods:** This prospective observational cohort study was conducted on consecutive critical ill children aged 1 month to 14 years old in 1 year. The blood sample was collected upon PICU admission. 25(OH)D deficiency was defined as<20 ng/mL. We performed univariate and multivariate analyses to evaluate associations with CV-SOFA and PRISM-III scores and other important outcomes.

**Results:** 296 critically ill children were enrolled in the study. The mean serum 25(OH)D level was 22.5 (IQR 16.3–31.8) ng/mL. The prevalence of 25(OH)D deficiency was 39.2% in critically ill children. 25(OH)D levels were significantly decreased in septic shock and associated with CV-SOFA and PRISM-III scores. In multivariate analysis, vitamin D deficiency is associated with CV—SOFA and PRISM—III scores.

**Conclusion:** 25(OH)D deficiency is prevalent in critically ill children at PICU admission and seems to be associated with higher CV-SOFA and PRISM-III scores. Our study provides additional data for 25 (OH) D statuses that impact the outcomes of critically ill children.

## Introduction

Vitamin D is known to be involved in a wide range of functions (1–4). Inadequate vitamin D status is common in many parts of the world. In adult critical illness, several studies have suggested that low vitamin D status is associated with adverse outcomes in septic shock (5, 6). Furthermore, some studies have suggested that vitamin D insufficiency is a risk factor in intensive care, and plays an essential role in infectious, immunologic, neurologic, cardiovascular, and respiratory disorders (7, 8). Nevertheless, these were based on studies for adult patients.

The prevalence of vitamin D deficiency in healthy children has also increased (9–13); however, most of the studies did not include younger children. There have although been several studies surveyed vitamin D levels in critically ill children (14–19), but these findings are often different and even contradictory. To date, the status and the effect of vitamin D deficiency on the outcome in critically ill children is still unclear, and the opinions on the importance of vitamin D deficiency in critically ill children remains controversial. Therefore, more studies and evidence from the different critical ill pediatric population are mandatory to address this dilemma.

We have previously studied the vitamin D status in severe hand-foot-and-mouth disease and found that children with this disease have very low vitamin D levels, which was associated with a poor prognosis (20). Therefore, we expanded the study to survey all critical illnesses in the pediatric intensive care unit (PICU) and its relationship with disease severity.

With this background, the purpose of this study was to examine the association between vitamin D deficiency and main clinical characteristics that represent the severity of the disease and outcome in children with critical medical illness admitted to our PICU. We hypothesized that the incidence of vitamin D deficiency in children in PICU was high, and correlated with the cardiovascular sequential organ failure assessment and pediatric risk of mortality III scores in critically ill children.

## Materials and Methods

### Study Subjects

This was a prospective observational cohort study in PICU and Chongqing Key Laboratory of Child Health and Nutrition in the children's Hospital of Chongqing Medical University of China, a tertiary teaching hospital. The hospital institutional review board approved the study [Approval No.: (2015) Ethics Review (Research) No. (92)], which was registered at the Chinese Clinical Trial Registry (http://www.chictr.org.cn/showprojen.aspx?proj=12027; Registration No.: ChiCTR-OOC-15007152).

### Inclusion and Exclusion Criteria

We screened all consecutive patients admitted to PICU from September 2016 to August 2017. Patients who had an estimated PICU stay of more than 48 h and were aged from 1 month to 14 years old were deemed eligible to participate and were enrolled within the first 24 hours of the PICU admission. Exclusion criteria included patients (1) admitted to PICU for postoperative monitoring, or (2) underwent blood purification, or (3) were taking isoniazid and rifampicin to treat tuberculosis and, or had epilepsy and had taken antiepileptic drugs (21) within the last 1 week. Parents or surrogates provided written informed consent. They were then interviewed via a detailed questionnaire on their child's vitamin D supplementation over the last 7 days, which including their intake of vitamin D, vitamin D-fortified formula, or any other vitamin D supplements. The conclusion of questions was dichotomized as yes or no to account for inconsistencies in the daily intake amount between patients. We defined “yes” as a total amount of any forms of vitamin D supplement per day of ≥400 IU for children ≤1-year-old or ≥600 IU for children > 1-year-old.

### Data Collection

Blood specimens (3 mL each) were obtained from the subjects as early as possible upon PICU admission (always within 12 h), before both treatment and enteral and/or parenteral nutrition. Previously obtained and stored excess laboratory samples were retrieved from the clinical laboratory for some patients as we were not able to obtain consent at the time of PICU admission. Each blood specimen was collected in lithium heparin tubes and harvested by centrifugation at 3,000 g for 10 min to obtain 1.5–2 mL of serum, then frozen at −80°C and sent in batches to a nationally accredited third party for the quantitative analysis of 25(OH)D. The main information collected from all subjects included gender, age, weight and height (W/H or BMI z-score), primary diagnosis categories, and the presence of any chronic disease conditions, infections, or mechanical ventilation. Severity of illness in the first 12 h was measured by using the pediatric risk of mortality III (PRISM-III) score. This was independently evaluated by two attending physicians, and the results were averaged. The cardiovascular sequential organ failure assessment (CV-SOFA) score was used to assess the maximum level of vasopressin use during PICU admission (22), with 0–1: no vasopressors; 2: dopamine ≤5 μg/kg/min; 3: dopamine 5–15 μg/kg/min or norepinephrine/epinephrine ≤0.1 μg/kg/min; and 4: dopamine >15 μg/kg/min or norepinephrine/epinephrine >0.1 μg/kg/min. The PRISM-III and CV-SOFA scorers were unaware of the 25(OH)D levels. All patients were followed-up for 30 days, with the 30-day outcome as the endpoint. Patient survival and death were recorded.

To determine the infection status at admission, patients who had any cultured or viral test on the day of admission to PICU, or who were diagnosed with confirmed or suspected infection within 7 days prior to PICU admission were reviewed by the PICU physician. Confirmed infection was defined as the culture of life-threatening pathogenic bacteria, fungal or viral pathogen, from the blood, cerebrospinal, pleural, or peritoneal fluid deep sputum (via fiber-bronchoscope), or urine (via indwelling catheter). Suspected infections included all patients who met the criteria for systemic inflammatory response syndrome, community-acquired pneumonia, or gastroenteritis, but tested negative for microorganisms and received a course of antibiotics. Septic shock was defined as a patient with a confirmed or suspected infection and on vasopressor therapy (CV-SOFA score ≥3) at PICU admission. All data were managed using an electronic data collection form. Mechanical ventilation (MV) in PICU was defined as any form of mechanical ventilation for a total of more than 24 h.

### Measurement Method and Range of Reference Values

The serum 25(OH)D was analyzed using liquid chromatography–tandem mass spectrometry (18). The pretreatment method of liquid–liquid extraction was used in the project, including a quality control. The experimental standard and internal standard were purchased from Sigma (St. Louis, MO, USA). The quality control was purchased from RECIPE (Munich, Germany). In order to ensure the accuracy of the method, we used the standard substance SRM972 (from American Standards Committee) to verify the accuracy of the 25(OH)D detection method. As defined by the *Chinese Medical Association* (21, 23), *the New England Journal of Medicine* (1) and *Global Consensus Recommendations on Prevention and Management of Nutritional Rickets* (24), the serum 25(OH)D concentrations were classified as follows: >20 ng/mL, normal; 15–20 ng/mL, relative deficiency; and <15 ng/mL, absolute deficiency. In this study, 25(OH)D deficiency was defined as ≤20 ng/mL.

### Statistical Methods

Data were analyzed using SPSS version 20.0 (IBM Corp., Armonk, NY, USA). The Kolmogorov–Smirnov one sample test was used to test whether variables were normally distributed. The abnormally distributed variables are expressed as median [interquartile range (IQR), or 95% confidence interval (CI)] as appropriate. Categorical variables are presented as counts and percentages. Spearman's correlation coefficient was used to assess the association of 25(OH)D with other variables, the Mann–Whitney test for dichotomous variables, and the Kruskal–Wallis test for multicategorical variables as most variables were not distributed normally. Frequencies of events were compared using a χ^2^ test. Logistic regression models that were predefined in the study protocol were fitted and adjusted for the main prognostic factors. We used multivariable logistic regressions to assess the influence of risk factors on vitamin D deficiency, CV-SOFA, and PRISM-III scores. Patient characteristics associated with 25(OH)D in univariate analysis (*P* ≤ 0.10) or potential predictors were included in the multivariable models. Results from the regression were reported as adjusted odds ratios (OR) and corresponding 95% CIs. *P* < 0.05 in a two-tailed test was taken as criteria for being considered statistically significant. All figures in this study were produced using Medcalc 18.2 software (Ostend, Belgium).

## Results

### General Condition of the Selected Subjects

**Figure 1:**
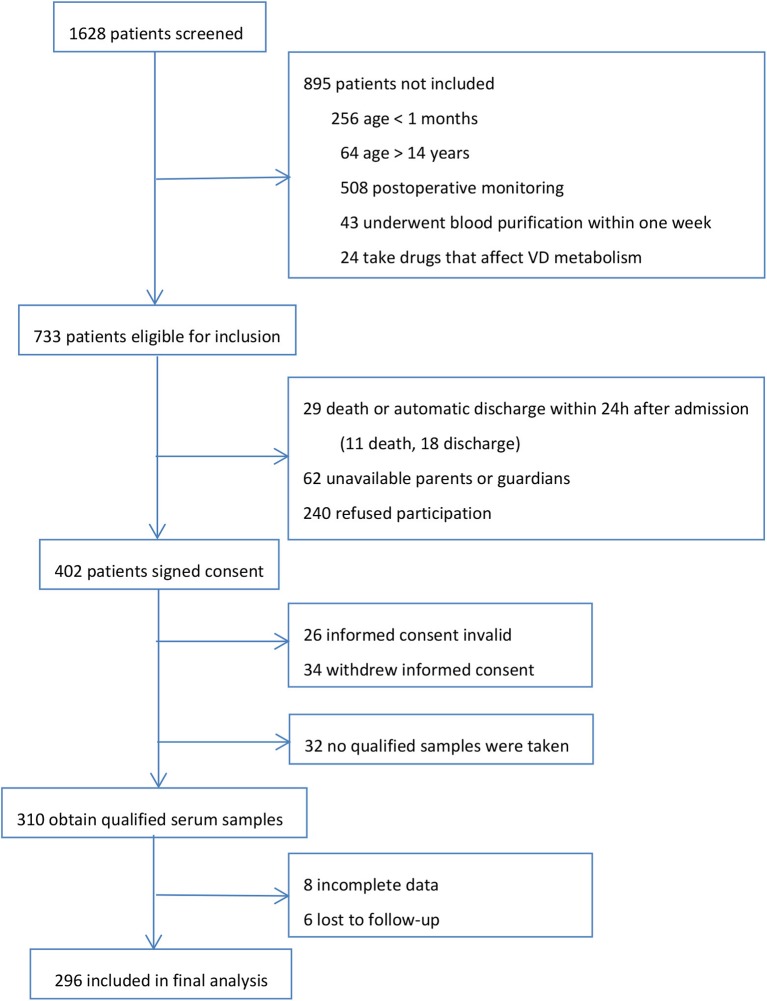
Research profile.

During the 1 year study, we screened all 1,628 patients admitted to PICU. Our final evaluable cohort consisted of 296 patients with valid test results and data. The research profile and reasons for not enrolling subjects are shown in Figure 1. There were no apparent differences in the demographics and admission PRISM-III scores between those who were eligible for inclusion but not included in the final analysis (*n* = 437) and those who were finally included for analysis in the study cohort.

### Distribution and Variation of 25(OH)D

**Figure 2:**
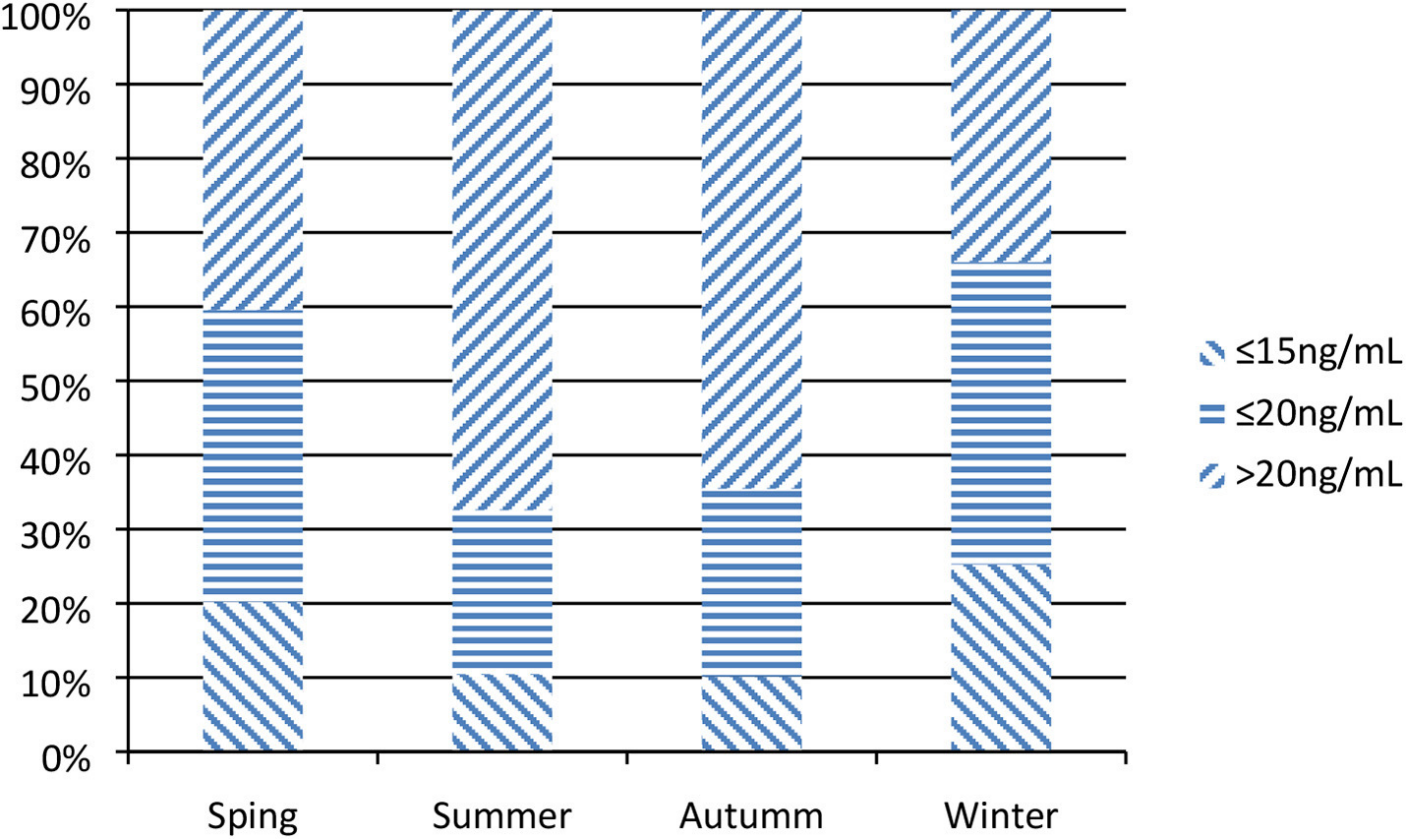
Seasonal variation of vitamin D in critically ill children.

The median 25(OH)D level of enrolled patients was 22.5 ng/mL (IQR 16.3, 31.8); 50.1% had a 25(OH)D level ≤30 ng/mL (*n* = 150), 39.2% had one ≤20 ng/mL (*n* = 116), and 18.9% had one ≤15 ng/mL (*n* = 61). In winter and spring [(Nov-Apr) (18.5 (13.6, 25.8) ng/mL] were obviously lower than those observed in summer and autumn [(May-Oct) 26.0 (19.7, 35.8) ng/mL) (Figure 2).

### Relationship Between 25(OH)D Levels and Demographic and Clinical Characteristics

Table 1 shows the baseline demographic and clinical characteristics of the 296 patients. In the univariate analysis and comparison stratified by serum 25(OH)D levels, using 20 ng/mL as the cutoff point, vitamin D deficiency was associated with lower W/H or BMI z-score, admitted in spring or winter, no vitamin D supplementation, suffering confirmed or suspected infection, higher CV-SOFA and PRISM-III scores, higher blood D-Dime, lactate, and procalcitonin levels, higher incidence of mechanical ventilation and 30 days mortality after PICU admission(Table 2).

**Table 1:** Baseline demographic and clinical characteristics of subjects.

**Variable**	***N* = 296**
Male (*n*, %)	171 (57.8)
Age, month (median, IQR)	20.5 (7, 56.5)
≤12 months (*n*, %)	109 (36.8)
12–36 months (*n*, %)	91 (30.7)
>36 months (*n*, %)	96 (32.4)
z-score (W/H, BMI) (median, IQR)	−0.65 (−1, 8, 0.5)
z-score < −2 (n, %)	58 (19.6)
PICU admission seasons in Spring or Winter (n, %)	162 (54.7)
Additional vitamin D supplementation (n, %)	198 (66.9)
Underlying chronic conditions (n, %)	97 (26.0)
Need for Mechanical ventilation (*n*, %)	128 (43.2)
Any confirmed or suspected infection (*n*, %)	182 (61.5)
CV-SOFA score (median, IQR)	1 (0, 3)
CV-SOFA≥3 (*n*, %)	76 (25.8)
PRISM-III score (median, IQR)	14 (9, 18)
PRISM-III≥10 (*n*, %)	220 (74.3)
Duration of PICU stay(median, IQR)	6 (4.9)
Died in 30 days after PICU admission (*n*, %)	39 (13.1)
**Primary diagnostic categories (*****n*****, %)**	
Acute poisoning and trauma	42 (14.2)
Respiratory	52 (17.6)
Neurological	34 (11.5)
Digestive	42 (14.2)
Circulatory	63 (21.3)
Endocrine, immune,e and metabolic	38 (12.8)
Hematologic, cancer and others	25 (8.4)

*CV-SOFA: cardiovascular sequential organ failure assessment; PRISM-III: pediatric risk of mortality III*.

**Table 2:** Demographic and Clinical features stratified by serum 25(OH)D status.

	**≤20 ng/mL (*n* = 116)**	**>20 ng/mL (*n* = 180)**	***P***
Male (*n*, %)	67 (57.8%)	99 (55.0%)	0.997
Age, month(IQR)	21.5 (7, 52.5)	19 (7,58)	0.792
z < −2 (*n*, %)	30 (25.9%)	28 (15.6%)	0.019
Spring or Winter PICU admission (*n*, %)	77 (66.4%)	71 (41.1%)	<0.001
Vitamin D supplementation (*n*, %)	23 (19.8%)	75 (41.7%)	0.001
Underlying chronic conditions (*n*, %)	81 (69.8%)	117 (65%)	0.389
Any confirmed or suspected infection (*n*, %)	79 (68.1%)	101 (56.1%)	0.023
CV-SOFA score(IQR)	2 (1, 3)	1 (0, 2)	0.005
CV-SOFA score≥3 (*n*, %)	40 (34.5%)	36 (20%)	0.006
PRISM-III score(IQR)	16 (12,19)	11 (6, 16)	<0.001
PRISM-III score≥10 (*n*, %)	105 (90.5%)	115 (63.9%)	<0.001
D-Dimer(mg/L) (IQR)	2.1 (0.69, 6.38)	0.9 (0.49, 2, 78)	0.001
Lactate(mmol/L) (IQR)	1.6 (1.15, 3.6)	1.1 (0.7, 1.9)	0000
procalcitonin(μg/L) (IQR)	8.6 (4, 14.2)	6.4 (3.6, 10.4)	0.026
Mechanical ventilation (*n*, %)	97 (83.6%)	131 (72.7%)	0.045
30 days Mortality (*n*, %)	22 (19%)	17 (9.4%)	0.008

*Testing for association with serum 25(OH)D level by Mann–Whitney test (dichotomy) or chi-square test. CV-SOFA: cardiovascular sequential organ failure assessment; PRISM-III: pediatric risk of mortality III*.

### Infection Upon PICU Admission and Association With 25(OH)D Levels

Although patients with confirmed or suspected infection had lower 25(OH)D levels, there was no significant difference between patients with confirmed positive viral or bacterial infections and other infectious patients. However, 25(OH)D levels were markedly lower in the 42 patients with septic shock than other confirmed or suspected infectious (Table 3).

**Table 3:** Infection on PICU admission and association with 25(OH)D levels.

**Infection on PICU admissiona^**a**^**	**N**	**25(OH)D(ng/mL) M(IQR)**	***P***
Confirmed positive viral test	44	21.9 (13.0, 30.1)	0.847^b^
Confirmed positive bacterial test	47	21.5 (14.5, 25.1)	0.644^a^
Any confirmed microbiologic test	88	22.0 (15.3, 28.8)	0.389^c^
Septic shock^d^	42	18.9 (13.3, 23.9)	0.033^e^

a Includes confirmed but non-life-threatening infection.

b vs. other confirmed or suspected infectious.

c vs. suspected infectious with no microbiologic confirmation.

d Cardiovascular sequential organ failure score ≥ 3 plus confirmed bacterial (n = 7), fungal (n = 1), viral infection(n = 10), multiple (n = 2) or suspected infection (n = 22).

e *vs. other confirmed or suspected infections without septic shock*.

### Relationship Between 25(OH)D Levels and Illness Severity

Thirty-nine (13.2%) patients died within 30 days of PICU admission (27 died while in the PICU), with a median 25(OH)D level of 19.3 ng/mL (IQR 8.7–25.8). Logistic regression analysis showed that vitamin D was not determined to be associated with mortality (Table 4, model 1).

**Table 4:** Multivariable logistic regression models assessing the variables associated with 30-days Mortality, CV-SOFA, and PRISM-III score.

**1. 30-days Mortality**	**Adjusted OR(95%CI)**	***P***
Gender(female vs. male)	1.956 (0.917–4.171)	0.082
Age(per 1 month increase)	0.997 (0.989–1.006)	0.513
z-score (per 1 increase)	0.696 (0.522–0.927)	0.013
Vit D Supplements (yes vs.no)	1.271 (0.542–2.98)	0.581
Confirmed or suspected infections (yes vs.no)	2.004 (0.74–5.428)	0.172
Underlying chronic illnesses (yes vs.no)	1.069 (0.473–2.416)	0.872
Winter and spring (yes vs.no)	0.528 (0.234–1.19)	0.124
Procalcitonin(per 1 μg/L increase)	1.053 (1.022–1.085)	0.001
Lactate(per 1 mmol/L increase)	1.106 (0.98–1.248)	0.101
D-Dimer(per 1 mg/L increase)	1.003 (0.971–1.035)	0.873
Serum 25(OH)D(≤20 ng/mL vs.>20 ng/mL)	1.316 (0.585–2.962)	0.506
**2. CV-SOFA ≥3**		
Gender(female vs. male)	0.848 (0.459–1.566)	0.599
Age(per 1 month increase)	0.998 (0.991–1.005)	0.590
z-score (per 1 increase)	0.847 (0.675–1.065)	0.155
Vit D Supplements (yes vs.no)	1.559 (0.795–3.058)	0.196
Confirmed or suspected infections (yes vs.no)	7.164 (3.246–15.808)	<0.001
Underlying chronic illnesses (yes vs.no)	1.887 (0.964–3.695)	0.064
Winter and spring (yes vs.no)	0.787(0.418–1.483)	0.459
Procalcitonin(per 1 μg/L increase)	1.161 (1.098–1.229)	<0.001
Lactate(per 1 mmol/L increase)	1.067 (0.95–1.197)	0.275
D-Dimer(per 1 mg/L increase)	1.029 (1.005–1.054)	0.017
Serum 25(OH)D(≤20 ng/mL vs.>20 ng/mL)	2.106 (1.076–4.124)	0.030
**3. PRISM ≥10**		
Gender(female vs. male)	0.681(0.387–1.198)	0.183
Age(per 1 month increase)	1.001(0.994–1.007)	0.835
z-score (per 1 increase)	0.884(0.722–1.083)	0.235
Vit D Supplements (yes vs.no)	1.166 (0.641–2.118)	0.615
Confirmed or suspected infections (yes vs.no)	1.03 (0.561–1.891)	0.924
Underlying chronic illnesses (yes vs.no)	0.833 (0.455–1.526)	0.555
Winter and spring (yes vs.no)	1.019 (0.574–1.809)	0.950
Procalcitonin(per 1 μg/L increase)	1.025 (0.986–1.066)	0.214
Lactate(per 1 mmol/L increase)	1.021 (0.891–1.17)	0.760
D-Dimer(per 1 mg/L increase)	1.016 (0.985–1.049)	0.317
Serum 25(OH)D(≤20 ng/mL vs.>20 ng/mL)	4.94 (2.345–10.407)	<0.001

*CV-SOFA: cardiovascular sequential organ failure assessment; PRISM-III: pediatric risk of mortality III. Hosmer and Lemeshow test: 30-days Mortality, p = 0.416; CV-SOFA score, p = 0.570; PRISM-III score, P = 0.595*.

**Figure 3:**
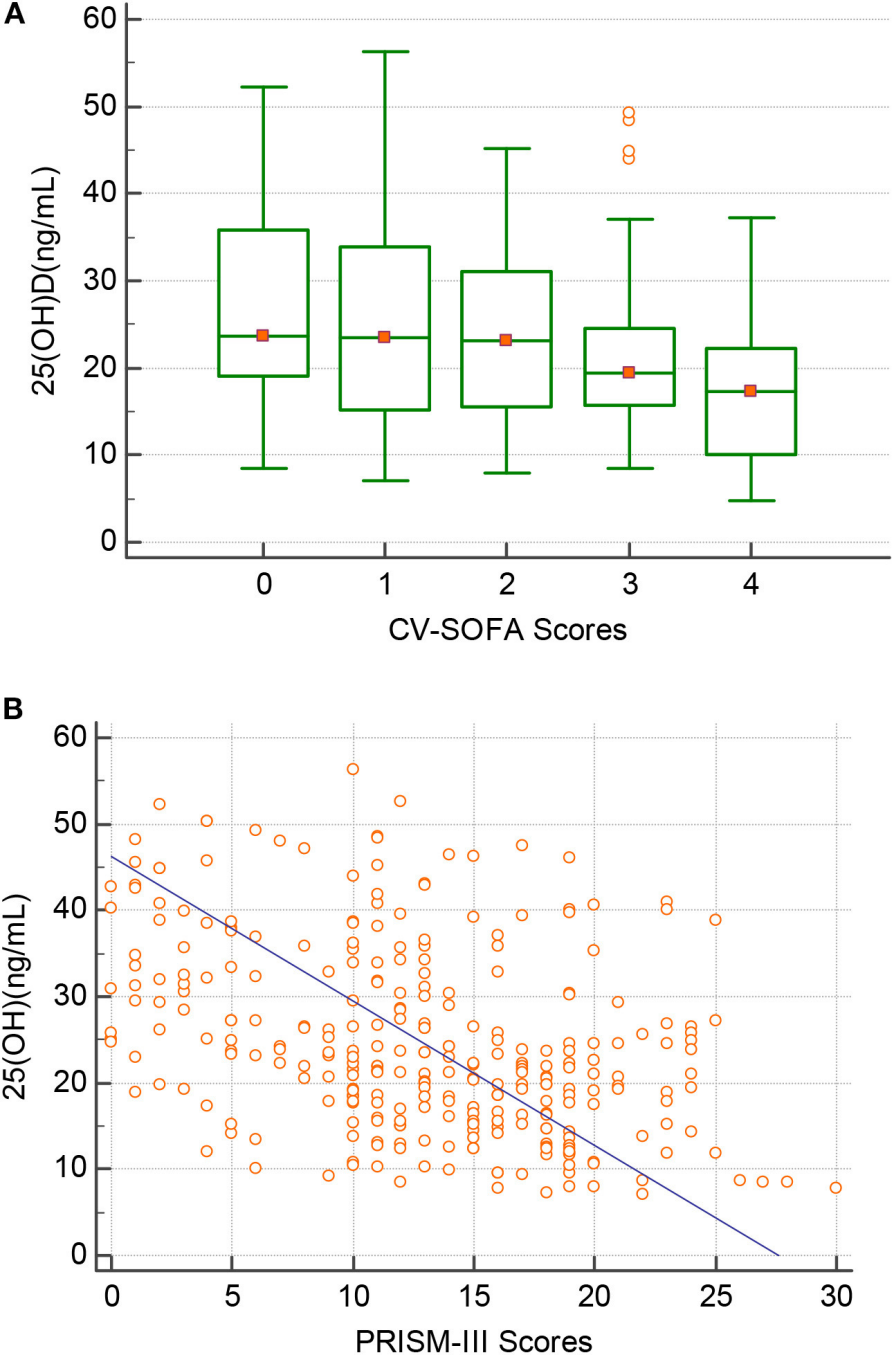
Correlation between the 25(OH)D level(Y-axis) and **(A)** the maximum cardiovascular SOFA (CV-SOFA) (X-axis) (*r* = −0.193, *P* = 0.0008) during the ICU stay and **(B)** the PRISM-III score(X-axis) (*r* = −0.397, *P* = 0.0001) at PICU admission in critically ill children.

There were 76 patients (25.7%) who received more vasopressors (CV-SOFA ≥ 3) during their PICU stay. These patients had significantly lower 25(OH)D levels. Increasing vasopressor use (CV-SOFA score) was correlated with lower 25(OH)D levels (*r* = −0.193, *P* = 0.0008; Figure 3A) on PICU admission. In the multivariable logistic regression model (Table 4, model 2), lower 25(OH)D levels, or confirmed or suspected infections, were independently associated with a higher CV-SOFA score.

The raw median PRISM-III score at PICU admission was 12 (IQR 7–18) and was stronger inversely correlated with the 25(OH)D level (*r* = −0.397, *P* = 0.0001; Figure 3B). A multivariable logistic regression model was created for assessing the variables associated with the PRISM-III score. After adjusting for the associated factors, as shown in Table 4, model 3, lower admission 25(OH)D levels were independently associated with PRISM-III scores.

## Discussion

Vitamin D deficiency is considered a global public health problem with a high prevalence in children and adolescents (25). Circulating 25-hydroxy D (25(OH)D) is the accepted marker for evaluating vitamin D status (8). In this prospective cohort study, we found a high incidence of vitamin D deficiency in critically ill children aged 1 month to 14 years, 25(OH) D levels were inversely associated CV-SOFA and PRISM-III score on PICU admission. Since the admission dates of subjects included in this study were equally distributed across all seasons, we observed an evident seasonal and monthly variation in 25(OH)D serum levels. The median serum 25(OH)D level in winter and spring were lower than that in other seasons, especially from January to March.

We found that the median 25(OH)D levels in critically ill children were significantly lower than those found for healthy children that we had previously investigated within the same area (28.1 ng/mL) (20) as well as in the northeast of China (26.3 μg/L), investigated by Chen (11). The 39.2% prevalence of vitamin D deficiency (38.5% in infants, 37.4% in toddlers, and 41.7% in school-aged children and adolescents) in our cohort of critically ill children was lower than that found in a previous study (69%) by Asilioglu N [(14), but more similar to a previous study (40%) by Madden (17).

Lower W/H or BMI z-scores were associated with lower 25(OH)D levels. It was reported that vitamin D is lower in obese children (26), but only four children with a z-score > 2 and they had >20 ng/mL vitamin D in our study cohort. The difference in conditions between healthy and severely ill children may be the reason for the discrepancy in 25(OH)D levels as compared with previous studies. In our study, vitamin D or vitamin D-fortified formula supplementation before PICU admission had a huge impact on effectively preventing 25(OH)D deficiency in critically ill children, especially in cold seasons when sunlight exposure was limited.

A previous study found that metabolomics profiles are significantly different in critically ill patients with a 25(OH)D level ≤15 ng/mL relative to those with levels >15 ng/mL (27). However, in this study, there was no significant difference in 25(OH)D levels between the different primary diagnostic categories at PICU admission, except for acute poisoning and trauma, and digestive diseases. Because of the limited number of patients, we did not perform subgroup analysis.

Many studies have suggested that children with infections may have lower vitamin D levels, and that vitamin D deficiency prior to hospitalization is associated with greater mortality and a greater prevalence of bacteria-positive blood cultures in critically ill patients (28, 29). In our study, children with confirmed or suspected life-threatening infections showed lower 25(OH)D levels compared to patients without infection, but no difference among the different causes of infections. However, those with septic shock had significantly lower levels of 25(OH)D, which was closely related to higher CV-SOFA scores (*r* = −0.206, *p* < 0.001). Systematic analysis of the relationship between vitamin D and septic shock has not been previously conducted, though the earlier study found that septic shock patients with extremely low vitamin D levels needed vasopressor support for a more extended period (30). Another previous study also indicated that vitamin D deficiency affects the catecholamine system and exacerbates the instability of the cardiovascular system (31).

The exact relationship between vitamin D and septic shock is yet to be understood; however, the low vitamin D levels in sepsis patients may be ascribed to protein catabolism, which reduces the level of vitamin D binding protein, a critical protein that is a predictor of mortality in ICU and is associated with the early stage of sepsis and a poor prognosis when its absolute levels decline (29). On the other hand, the known effects of 25(OH)D include inhibition of T cell proliferation, regulation of pro-inflammatory and anti-inflammatory cytokines (32, 33), and 25(OH)D deficiency maybe also contributes to the progression of sepsis shock. Also, many children with septic shock were given large boluses of fluid in the early stages as fluid resuscitation, which caused blood dilution and may also partly explain the decreased vitamin D levels observed in children with septic shock.

We also investigated whether higher CV-SOFA and PRISM-III scores were associated with lower 25(OH)D levels. By evaluating the relationship using regression approaches, a statistically significant inverse relationship between 25(OH)D levels and CV-SOFA or PRISM-III was reported. These results suggest that lower 25(OH)D levels are likely to be linked to disease severity and play a role in promoting disease progression. The level of 25(OH)D may help in evaluating critical illness, and monitoring serum 25(OH)D concentrations in children in PICU may assist in determining the progression and prognosis of critical disease.

Some previous studies found that vitamin D deficiency was associated with increased mortality, while others did not (19). According to the present study, we found that patients who died within 30 days of PICU admission had significantly lower 25(OH)D levels compared to survivors. However, in the multivariable regression analysis, after adjustment for gender, age, z-score, vitamin D supplementation, season, and some factors associated with disease severity, 25(OH)D deficiency was not correlated with mortality.

It worth noting that a previous study showed that there was no new evidence that vitamin D supplementation could affect most non-skeletal conditions (34). In our study, the use of logistic regressions also did not show any effect of prior vitamin D supplementation on CV-SOFA and PRISM-III scores. Therefore, it is still necessary to determine whether vitamin D supplementation in the early stages of critical illness can improve clinical outcomes.

In the present study, we identified a high prevalence of vitamin D deficiency in critically ill children admitted to the PICU, and an inverse association between 25(OH)D levels and CV-SOFA and PRISM-III score on admission, which provides additional prognostic information in children with critical illnesses. Those patients who refused to participate and withdrew informed consent were unlikely to have differed from the enrolled subjects. Therefore, the samples in this study are likely to represent the most seriously ill pediatric patients. However, because of a lack of measurement of calcium and 1,25 OH form as well as lack of serial measurements of Vitamin D, and we were unable to assess thoroughly and follow up on the longitudinal trend in 25(OH)D levels throughout PICU. Therefore, extensive, multicenter studies are necessary to expand the sample size and extend the study period to further clarify the mechanism of different forms of vitamin D deficiency in the progression of critical illnesses.

## Data Availability Statement

The datasets generated for this study can be found in the https://data.mendeley.com/datasets/yd444n83cb/draft?a=7ba4f23e-a104-4764-aeac-4f60b457c2fe.

## Ethics Statement

The studies involving human participants were reviewed and approved by the review board of the children's Hospital of Chongqing Medical University of China. Written informed consent to participate in this study was provided by the participants' legal guardian/next of kin.

## Author Contributions

Conceptualization, writing-review, and editing: HD and FX. Data curation and investigation: JL. Formal analysis, funding acquisition, methodology, software, and writing-original draft: HD. Project administration: FX. Resources and visualization: CL. Supervision: FX. Validation: JL, CL, and FX.

### Conflict of Interest

The authors declare that the research was conducted in the absence of any commercial or financial relationships that could be construed as a potential conflict of interest.
